# DNA methylation exploration for ARDS: a multi-omics and multi-microarray interrelated analysis

**DOI:** 10.1186/s12967-019-2090-1

**Published:** 2019-10-17

**Authors:** Shi Zhang, Zongsheng Wu, Jianfeng Xie, Yi Yang, Lei Wang, Haibo Qiu

**Affiliations:** Department of Critical Care Medicine, Zhongda Hospital, School of Medicine, Southeast University, Nanjing, 210009 China

**Keywords:** ARDS, DNA methylation, mRNA, Multi-omics, Interrelated analysis

## Abstract

**Background:**

Despite advances in clinical management, there are currently no novel therapeutic targets for acute respiratory distress syndrome (ARDS). DNA methylation, as a reversible process involved in the development and progression of many diseases, would be used as potential therapeutic targets to improve the treatment strategies of ARDS. However, the meaningful DNA methylation sites associated with ARDS still remain largely unknown. We sought to determine the difference in DNA methylation between ARDS patients and healthy participants, and simultaneously, the feasible DNA methylation markers for potential therapeutic targets were also explored.

**Methods:**

Microarray data of human blood samples for ARDS and healthy participants up to June 2019 was searched in GEO database. The difference analyses between ARDS and healthy population were performed through limma R package, and furthermore, interrelated analyses of DNA methylation and transcript were accomplished by VennDiagram R package. Perl and sva R package were used to merge microarray data and decrease heterogeneities among different studies. The biological function of screened methylation sites and their regulating genes were annotated according to UniProt database and Pubmed database. GO term and KEGG pathway enrichment analyses were conducted using DAVID 6.8 and KOBAS 3.0. The meaningful DNA methylation markers to distinguish ARDS from healthy controls were explored through ROC (receiver operating characteristic curves) analyses.

**Results:**

Five datasets in GEO databases (one DNA methylation dataset, three mRNA datasets, and one mRNA dataset of healthy people) were enrolled in present analyses finally, and the series were GSE32707, GSE66890, GSE10474, GSE61672, and GSE67530. These databases included 99 patients with ARDS (within 48 h of onset) and 136 healthy participants. Difference analyses indicated 44,439 DNA methylation alterations and 29 difference mRNAs between ARDS and healthy controls. 40 methylation variations regulated transcription of 16 genes was explored via interrelated analysis. According to the functional annotations, 30 DNA methylation sites were related to the imbalance of inflammation or immunity, endothelial function, epithelial function and/or coagulation function. cg03341377, cg24310395, cg07830557 and cg08418670, with AUC up to 0.99, might be the meaningful characteristics with the highest performance to distinguish ARDS from healthy controls.

**Conclusions:**

44,439 DNA methylation alterations and 29 difference mRNAs exist between ARDS and healthy controls. 30 DNA methylation sites may regulate transcription of 10 genes, which take part in pathogenesis of ARDS. These findings could be intervention targets, with validation experiments to be warranted to assess these further.

## Background

Acute respiratory distress syndrome (ARDS) is a life-threatening form of respiratory failure that accounts for 10% of intensive care unit admissions. In spite of improvements in basic science and clinical research, treatment options for ARDS are still limited and the mortality of severe ARDS is 40–46% [1]. Most innovative therapies for ARDS have failed in the last decade, and clearly, there is a robust need for better insight in disease pathogenesis and subsequent emerging treatment strategies [1, 2].

The majority of studies focus on genomic or transcriptomic responses in ARDS [3–5]. Accumulating evidences demonstrate that the epigenetic alterations especial DNA methylation involved in the development and progression of many diseases, including various cancers, lupus, diabetes, asthma, and a variety of neurological disorders [6, 7]. It was reported that DNA methylation can directly block transcription by inhibiting the binding of specific transcription factors to their target sequences on the candidate gene, which could result in an obvious variation in transcriptome and lead to the occurrence and development of diseases eventually [8–10]. In this scenario, valuable DNA methylation variations could be used as biomarkers for molecular diagnosis of disease. Moreover, DNA methylation could also be potential target to explore new treatment strategy [6–10]. For instance, Dhas et al. showed that specific DNA methylation might be potential marker for prognosis of neonatal sepsis which may improve the treatment strategies [11].

To date, studies on DNA methylation about ARDS is insufficient. Szilagyi et al. showed that myosin light chain kinase (MYLK) epigenetic variations were implicated in ARDS pathogenesis and might influence ARDS mortality [12]. In this study, researchers only focus on single genetic variations and the other underlying methylated CpGs related genes still unclear. Fortunately, the author had uploaded the DNA methylation microarray data of patients with ARDS to the Gene Expression Omnibus (GEO) databases, which made it possible for us to explore DNA methylation in ARDS comprehensively. Beside these, there are three mRNA microarray data of patients with ARDS in GEO databases which provided by Kangelaris et al. [3], Dolinay et al. [4] and Howrylak et al. [5]. All of these public data will be helpful to us perform integrative analyses of DNA methylation and mRNA in ARDS.

In the current study, in order to find DNA methylation alterations and mRNA expression differences between patients with ARDS and health controls, we utilized difference analysis on DNA methylation microarray and mRNA microarray data. Furthermore, for exploration of the potentially meaningful DNA methylation alterations, we performed integrative analyses of DNA methylation variations and mRNA expression differences. In addition, to evaluate the value of these methylation alterations to distinguish ARDS, receiver operating characteristic curves (ROC) would be calculated.

## Methods

### Systematic search and data selection

**Fig. 1:**
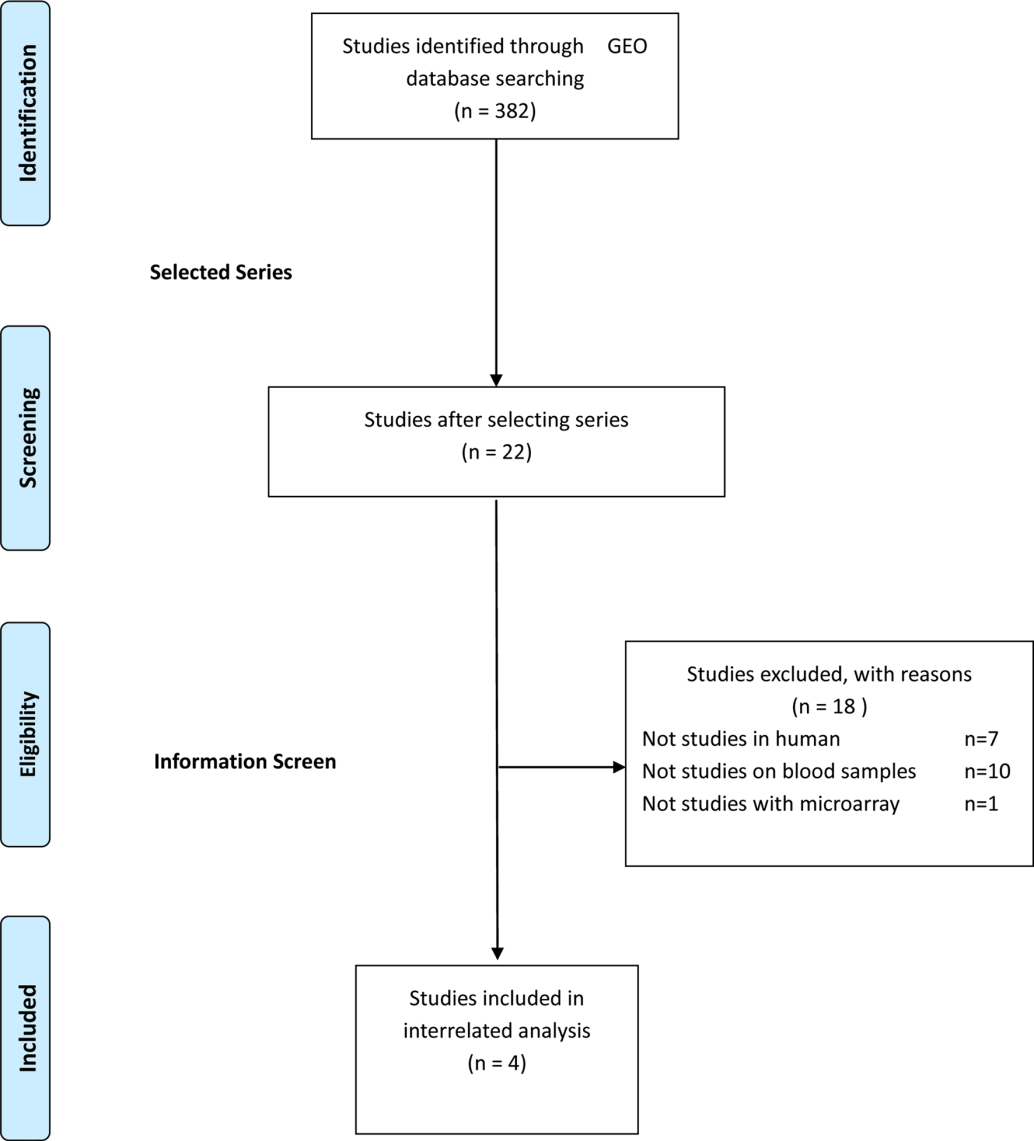
The flow-process diagram. The flow-process diagram on the left was how to screen microarray datasets

The GEO was searched for all expression microarray that matched terms of ARDS. Clinical studies of ARDS using peripheral blood of adult were retained. Datasets that utilized endotoxin or lipopolysaccharide infusion as vitro or animal models for ARDS were excluded. Clinical-gene expression microarray derived from sorted cells was also excluded (Fig. 1).

Expression microarray datasets of healthy participants were searched and set as the control group that matched terms of health and GPL10558 (the same platform number as included gene expression microarray of ARDS). Moreover, where longitudinal data were available for patients admitted with ARDS, we only included data derived from the within 48 h of onset.

### Gene expression normalization

ComBat normalization in the sva R package and Perl was used to co-normalize these cohorts into a single cohort, after re-normalizing from raw data. All datasets were downloaded as txt files, and DNA methylation microarray were re-normalized and batch corrected through minfi, impute, and wateRmelon R packages. Outputs from mRNA array were normal-exponential background corrected and then between-arrays quantile normalized using limma R package. For compatibility with microarray studies, expression was normalized using a weighted linear regression, and the estimated precision weights of each observation were then multiplied with the corresponding log2 to yield final gene expression values.

### Integrative analysis

There are two parts to the integrative analysis shown in Additional file 1: Figure S1: on the one hand, the differentially expressed DNA methylation sites and mRNAs were determined using the R package limma, which implements an empirical Bayesian approach to estimate gene expression changes using moderated t-tests. A log fold change > 0 was defined as hypermethylation or up-regulated genes, and a log fold change < 0 was defined as demethylation or down-regulated genes. For elimination of the influence of human species on DNA methylation, the intersection analyses of the DNA methylation alterations of the melanoderm and Caucasian groups were taken, Additional file 2: Figure S2.

On the other hand, the methylation sites with biological function and methylated genes were defined as hypermethylation accompanying with down-regulated genes and demethylation accompanying with up-regulated genes, simultaneously. In this scenario, we respectively took the intersection of hypermethylation sites and down-regulated genes, and the intersection of demethylation sites and up-regulated genes, Additional file 3: Figure S3.

The differentially expressed DNA methylation sites and mRNA were identified by significance criteria (adjusted P value < 0.05) as implemented in the R package limma. The intersection analyses were performed through VennDiagram R package.

### Functional annotation and enrichment of screened methylation alterations

Functional annotations and gene-annotation enrichment analyses of genes regulated by screened methylation variations were referenced the UniProt database, KOBAS 3.0 online database, DAVID 6.8 online database and PubMed database.

For the exploration of the interaction of screened genes, STRING online database was used to plot the protein–protein interaction network (PPI).

### Evaluation the value of methylation alterations to distinguish ARDS from healthy volunteers

To identify novel methylation alterations which would to distinguish ARDS from healthy volunteers, the receiver operating characteristic curves (ROCs) were performed to calculate the area under the curve (AUC) on screened DNA methylation variations using ROCR R package.

### Software and versions

Perl 64 was used to merge data; R x64 3.4.4 was conducted to process data, analyse data and plot diagrams; Cytoscape 3.6.1 was performed to plot network diagrams.

## Results

### Characteristics of the datasets

After search strategy and inclusive criteria, 5 studies containing one DNA methylation dataset (GSE67530) [12], 3 mRNA datasets of patients with ARDS (GSE32707, GSE66890 and GSE10474) [3–5], and one mRNA dataset of healthy people (GSE61672) [13] were used to build the methylation and mRNA expression profiling datasets, Table 1.

**Table 1:** Microarray data information

Information	Transcriptome ARDS 1	Transcriptome ARDS 2	Transcriptome ARDS 3	Transcriptome health	P	Genome ARDS	Genome health	P
Sample	18	29	13	106		39	30	
Series	GSE32707	GSE66890	GSE10474	GSE61672		GSE67530	GSE67530	
Platform	GPL10558	GPL6244	GPL571	GPL10558		GPL13534	GPL13534	
Year	2012	2015	2008	2015		2017	2017	
Country	USA	USA	USA	USA		USA	USA	
Contributor	Dolinay T	Kangelaris KN	Howrylak JA	Wingo AP		Zhang W	Zhang W	
Age: mean (SD)	54.0 (14.5)	59 (19)	56.2 (4.8)	50.91 (10.4)	0.88	52.5 (17.5)	58.2 (15.1)	0.67
Male%	46.4	55.0	46.0	41.5	0.89	59.0	46.7	0.44
Caucasian%	82.1	NA	84.0	59.4		46.15	50.0	
APACHE II score, mean (SD)	30.46 (9.0)^a^	116 (39)^b^	20.7(1.5)^a^			23.5(6.67)		
Direct lung injury %	50	72	38			100%		

^a^APACHE II score

^b^APACHE III score

DNA methylation microarray was the M-values (log2 ratio of the intensities of modified probe vs unmodified probe) of CpG probes (485,577 probes) that passed quality control and batch corrected. Gene microarray datasets were mRNA expression profiling after quality control and batch correction, with the median number of mRNA probes assayed as 25,128 (ranging from 22,277 to 47,220). Our integrated dataset included a total of 99 patients with ARDS and 136 health participants. The number of samples investigated ranged from 13 to 106 cases (median 30) across the studies. All datasets were from United States, and there was no statistical difference among datasets in age, and male.

### Screening of differentially expressed genes and methylation sites

**Fig. 2:**
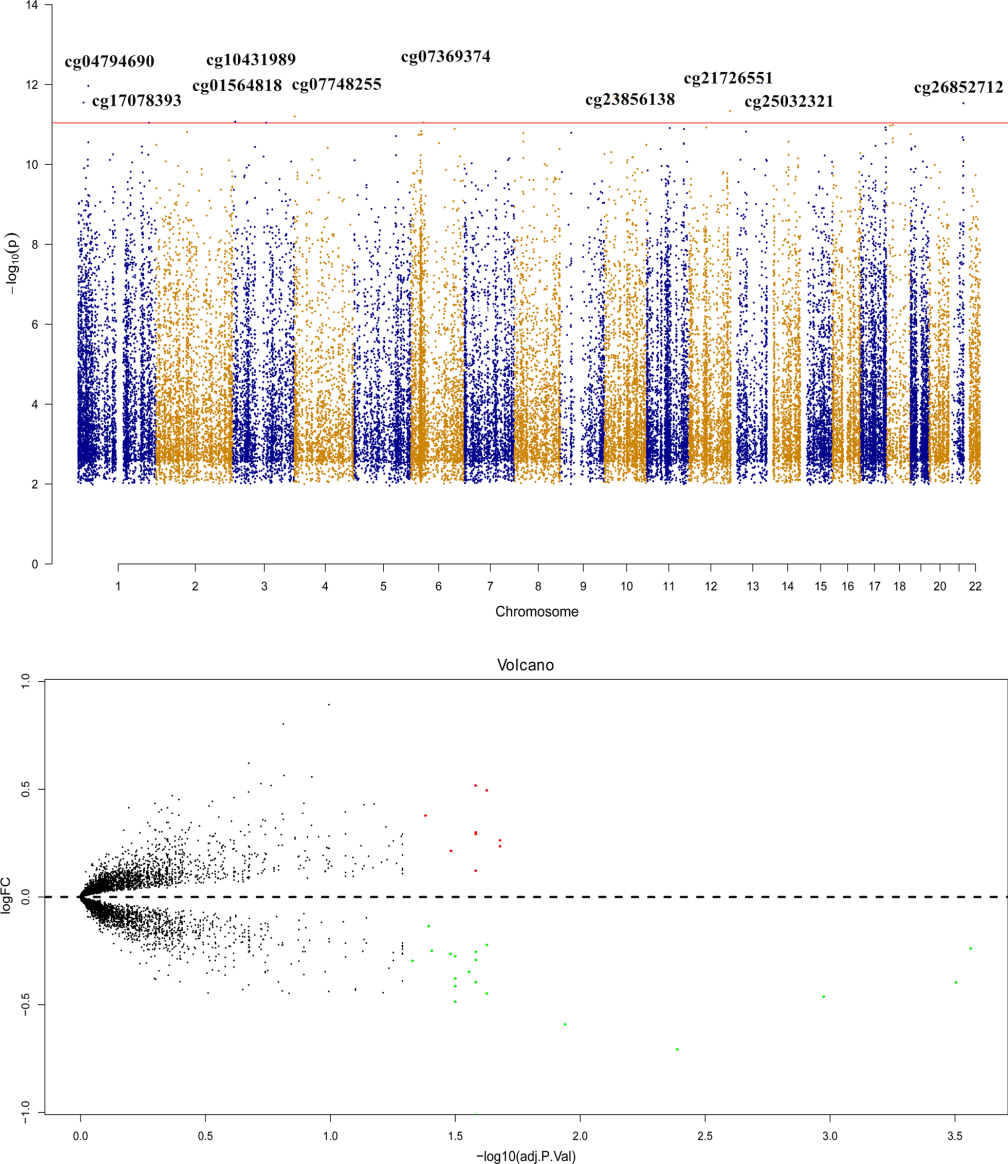
Differential methylation sites and mRNA expression. The Manhattan Figure at the top shows the differential methylation sites between the patients with ARDS and the healthy controls. The red line shows the 10 sites with minimum P values. The volcano figure at the bottom shows the mRNA expression. The red points indicate high-expression mRNA in ARDS, and the green points indicate low-expression mRNA according to the threshold of the FDR

In total, 22,654 hypermethylation sites and 21,785 demethylation sites were identified in Manhattan chart (Fig. 2). Sorted by adjusted P value from small to large, the top 10 methylation variations between patients with ARDS and healthy volunteers were cg17078393, cg04794690, cg01564818, cg07748255, cg07369374, cg23856138, cg21726551, cg25032321, cg10431989 and cg26852712. Obviously, 9 up-regulated mRNA and 20 down-regulated mRNA were identified in volcano plot (Fig. 2 and Additional file 4: Table S1).

### Exploration of methylation sites with biological function and methylated genes

**Fig. 3:**
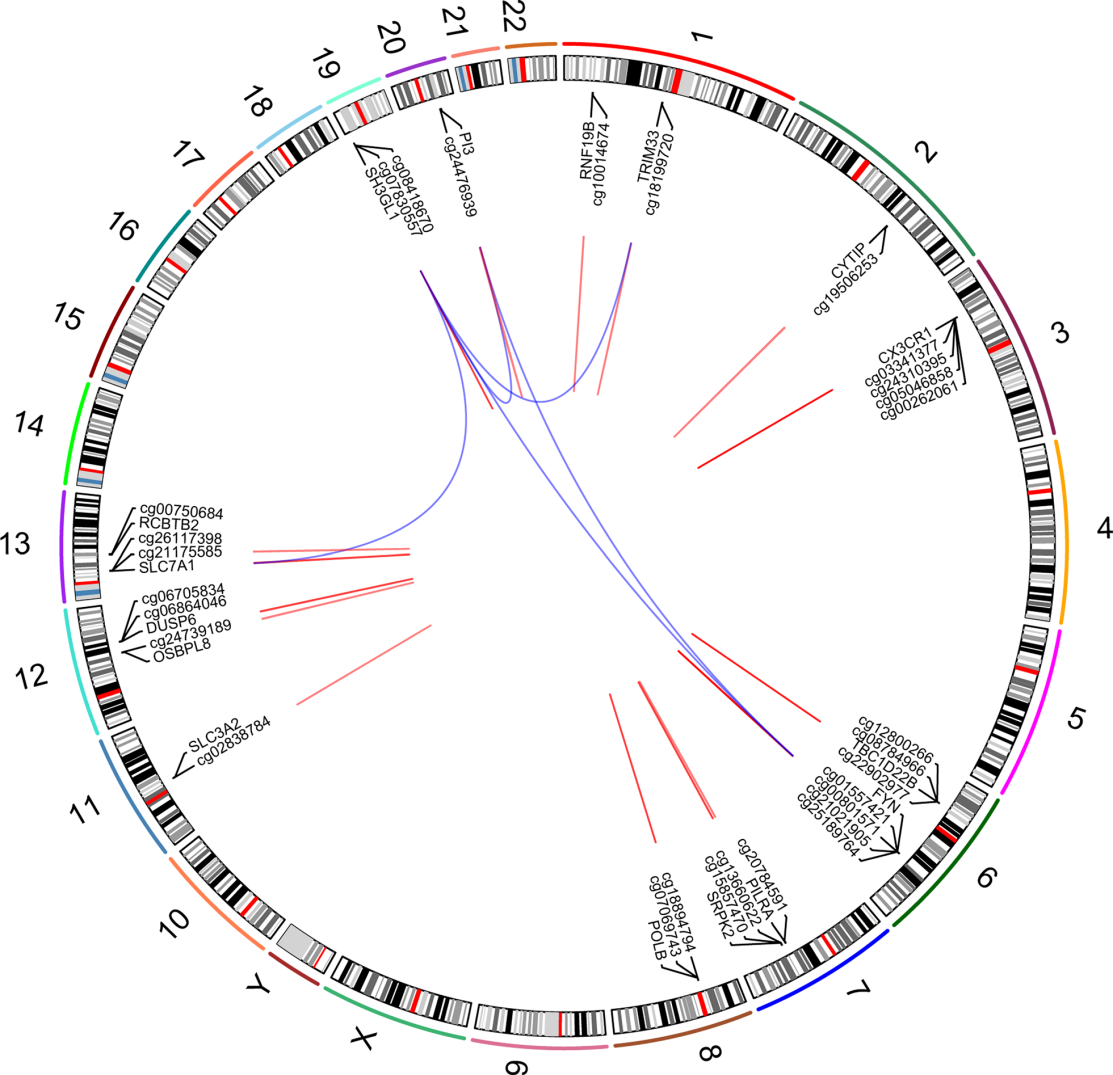
Circle diagram for the interrelated analysis and PPI analysis results. This diagram shows the positions of methylation sites and their target genes on 24 chromosomes after screening by preliminary interrelated analysis between DNA methylation and mRNA microarray datasets. The red lines represent the relationship between DNA methylation and their target genes. The blue lines represent interactions between proteins coded by these genes according to Protein Interaction Analyses

**Fig. 4:**
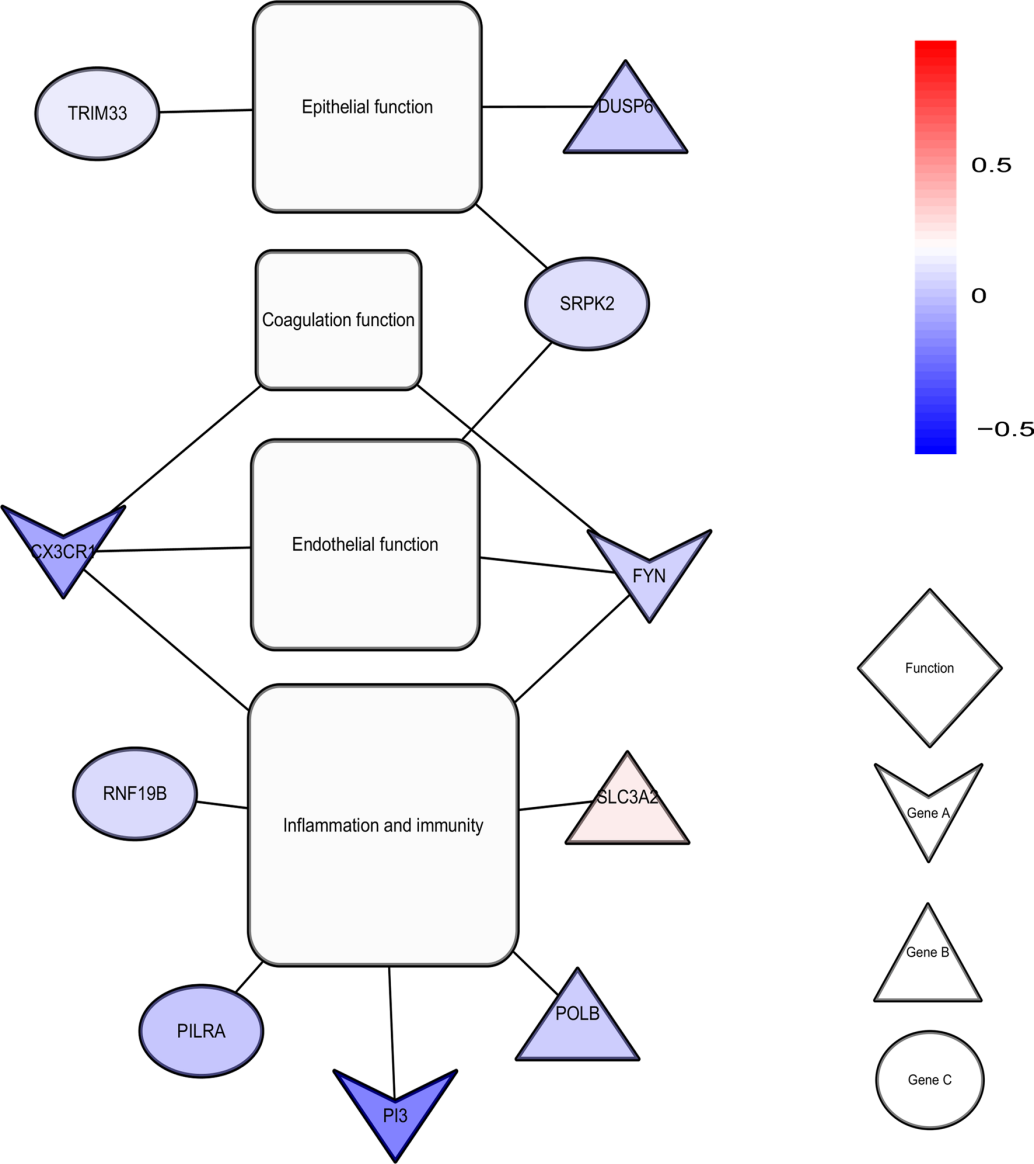
Network diagram for the relationship between the screened genes and the 4 mechanisms of ARDS. Colour depth represents the log-fold change of genes. Function represents the 4 mechanisms of ARDS. Genes in cluster A was confirmed to be related to the pathogenesis of ARDS in both ARDS animal models and in vitro experiments. Genes in cluster B was confirmed to be related to the pathogenesis of inflammation or immunity, endothelial function, epithelial function, and coagulation function in in vitro experiments. Genes in cluster C might be related to the above mechanism because these genes are related to immune regulation, angiogenesis and the cell cycle

16 genes were differentially methylated by interrelated analyses criteria, including 32 hypermethylation sites and 8 demethylation sites, demonstrated in Table 2 and Fig. 3. Overall, according to the function annotation, 30 methylation alterations were finally screened to be involved in the pathogenesis of ARDS through their potentially regulated 10 genes (Fig. 4), with the mRNA expression of 10 potentially regulated genes demonstrated in Additional file 5: Figure S4.

**Table 2:** Relationship between methylation sites and mRNA, with AUC of methylation sites

Low expressed mRNA	Hypermethylation sites	AUC to distinguish ARDS	High expressed mRNA	Demethylation sites	AUC to distinguish ARDS
CX3CR1	cg00262061	0.89	SH3GL1	cg07830557	0.99
CX3CR1	cg03341377	0.99	SH3GL1	cg08418670	0.99
CX3CR1	cg05046858	0.87	SLC3A2	cg02838784	0.79
CX3CR1	cg24310395	0.99	SLC7A1	cg21175585	0.83
CYTIP	cg19506253	0.97	SLC7A1	cg26117398	0.77
DUSP6	cg06705834	0.66	TBC1D22B	cg08784966	0.93
DUSP6	cg06864046	0.62	TBC1D22B	cg12800266	0.95
FYN	cg00801571	0.93	TBC1D22B	cg22902977	0.74
FYN	cg01557421	0.8			
FYN	cg02115050	0.7			
FYN	cg02755956	0.9			
FYN	cg02789394	0.98			
FYN	cg02816367	0.72			
FYN	cg04657000	0.94			
FYN	cg07725064	0.95			
FYN	cg11412876	0.61			
FYN	cg12636607	0.92			
FYN	cg17076443	0.6			
FYN	cg17587997	0.99			
FYN	cg20706496	0.93			
FYN	cg21021905	0.91			
FYN	cg25189764	0.91			
OSBPL8	cg24739189	0.83			
PI3	cg24476939	0.93			
PILRA	cg20784591	0.75			
POLB	cg07069743	0.49			
POLB	cg18894794	0.63			
RCBTB2	cg00750684	0.54			
RNF19B	cg10014674	0.55			
SRPK2	cg13660622	0.74			
SRPK2	cg15857470	0.93			
TRIM33	cg18199720	0.66			

*AUC* area under the receiver operating characteristic curves

Peptidase inhibitor 3 (PI3), C-X3-C motif chemokine receptor 1 (CX3CR1) and FYN proto-oncogene, Src family tyrosine kinase (FYN) have been confirmed to be related to the pathogenesis of inflammation or immunity, endothelial function, epithelial function, and coagulation function in both ARDS animal models and in vitro experiments. It was noteworthy that the mRNA level of PI3 in the plasma of patients with ARDS was significantly decreased.

The dual specificity phosphatase 6 (DUSP6), DNA polymerase beta (POLB); and solute carrier family 3 member 2 (SLC3A2) genes only had in vitro experimental evidence, and paired immunoglobin like type 2 receptor alpha (PILRA), SRSF protein kinase 2 (SRPK2), ring finger protein 1 (RNF1) and tripartite motif containing 33 (TRIM33) might be related to the pathogenesis of ARDS, as shown in Additional file 6: Table S2.

Gene Ontology (GO) and pathway enrichment analyses indicated that the 10 screened genes were enriched in 9 functions and 25 pathways, as shown in Additional file 7: Figure S5, Additional file 8: Figure S6. These functions and pathways included 2 types. The first type was related to endothelial and epithelial apoptosis and repair, including mTOR and MAPK pathways. The second type was related to inflammation or immunity, including adaptive immune response and leukocyte migration.

### Assessment of diagnostic efficacy on screened methylation sites

AUC was calculated on screened DNA methylation alterations based on ROC analyses, Table 2. The AUC on cg03341377, cg24310395, cg07830557 and cg08418670 were up to 0.99, which meant that these DNA methylation alterations could be most significant characteristics to distinguish ARDS from control subjects.

## Discussion

DNA methylation has been most studied and is classically associated with gene silencing via hypermethylation of CpG islands located in promoter regions of various diseases suppressor genes. In addition, DNA methylation is an attractive investigative tool for the study given that methylation is a reversible process. For instance, in myelodysplastic syndrome, demethylating agents have been tested in phase I clinical trials [14]. However, DNA methylation research on ARDS was inadequate and recent studies only focused on single genetic variations [15, 16]. Detailed studies of other novel candidates might lead to identification of unsuspected evolutionarily conserved mechanisms triggered by ARDS.

To our knowledge, this was the first study of all-encompassing analysis for DNA methylation alterations in ARDS, indicated in Additional file 9: Table S3. As furthermore research targets for improvement of therapies in ARDS, the present study uncovered 30 methylation alterations and their regulating DNA in ARDS regions. Meanwhile, as diagnostic molecules for ARDS, the clinicians might be interested in the top 10 difference methylation sites and 4 high diagnostic efficacy CpG islands variations.

Due to microarray experiments from human blood samples as the object of this study, 7 animal model studies and 10 studies not on blood samples were eliminated, leaving only 4 studies of microarray experiments and 1 study of transcriptome sequencing. In the consideration of comorbidity of hematologic malignancy existing in patients of transcriptome sequencing study, the sequencing data were eventually removed. Previous studies utilized only one single database, with a relatively small sample size. Transcriptome microarray datasets were not found healthy participants, which means in the need of searching healthy volunteers data. The data of healthy participants (generalized anxiety disorder score = 0) from one dataset related to anxiety disorder was extracted, according to matched terms of health and GPL10558 (the same platform number as included gene expression microarray of ARDS) in consideration of compatibility with microarray studies.

After merging these cohorts into a single cohort by Perl, batch correction and co-normalization were performed through ComBat normalization in the sva R package, which was a frequently-used method to decease heterogeneity among microarray studies [17–20]. In addition, the threshold values of screening differential mRNAs were P value < 0.05 in previous studies, which might lead to partially false positives. The significance criteria of adjusted P value < 0.05 aimed for reduction of false positives [21, 22]. Strikingly, it has been reported that the most epigenetic mutations appear unrelated to biological function [23].

Since DNA methylation could directly block transcription by inhibiting the binding of specific transcription factors to their target sequences on the candidate gene in the result of an obvious variation in transcriptome, integration with methylation data and matched transcriptome data was conducted to screen meaningful methylation mutations and methylated DNA regions [24].

Until now, the most significant 10 methylation variations found through difference analyses were lack of studies, with the function of their methylated gene related to immunity and cell proliferation, which could be appealing to researchers for further studies [25–29].

Tejera et al. [30] and Wang et al. [31] had confirmed that the mRNA level of PI3 in patients with ARDS was significantly decreased, which in turn might increase the activity of neutrophil elastase indirectly for PI3 plays a central role in controlling the excessive activity of neutrophil elastase. Liu and his colleagues [32] had showed that CX3CR1 was the crucial molecule that regulates the EGFR, Src and FAK pathways, which are crucial for epithelial and endothelial cell growth. The reduction of CX3CR1 in patients with ARDS could lead to damage to epithelial and endothelial cell regeneration. However, the mechanism of down-regulated PI3 and CX3CR1 in ARDS is unclear. Our study showed that the hypermethylation on cg24476939 of PI3 and 4 hypermethylation sites of CX3CR1 might explain down-regulation of PI3 and CX3CR1 in ARDS. Similar to PI3 and CX3CR1, FYN, DUSP6, POLB and SLC3A2, PILRA, SRPK2 and RNF1 were confirmed to be related to the pathogenesis of ARDS, including inflammation or immunity, endothelial–epithelial barrier, and coagulation function [33–36]. The present study verified above variations on mRNA levels in ARDS. Moreover, methylation alterations in these DNA regions of molecules might be responsible for the mRNA level variation in ARDS, which might be potential therapeutic targets for ARDS.

In addition, the ROC analyses on screened methylation alterations indicated that the AUC on cg03341377, cg24310395, cg07830557 and cg08418670 was up to 0.99, which meant that these DNA methylation alterations were valuable targets to distinguish ARDS from healthy controls, Table 2.

There are several limitations to the present study. First, as a retrospective study of primarily publically available data, we are not able to control for demographics, infection, patient severity, or individual treatment, and there was no analysis combined with clinical data because the microarray data did not provide clinical information. Second, although 4 microarray datasets were merged, a larger sample size may have been better. The ideal method of transcriptome and genome interrelated analysis should measure DNA methylation and mRNA in the same samples and then perform a co-expression analysis. However, in the public database, there was no such completed microarray data regarding ARDS. Besides, for the limitation of bioinformatics, the further studies and experiments should be conducted to sequentially verify and research these methylation alterations screened by our analyses.

## Supplementary information

**Additional file 1: Figure S1.** The flow-process diagram of the integrative analysis for exploration of the potentially meaningful DNA methylation alterations.

**Additional file 2: Figure S2.** The Venn diagram for the intersection of methylation sites between Melanoderm and Caucasian. The left Venn diagram was the intersection of hypermethylation sites between Melanoderm and Caucasian. The right Venn diagram was the intersection of demethylation sites between Melanoderm and Caucasian.

**Additional file 3: Figure S3.** The Venn Diagram for the intersection of methylation alterations and mRNA variations. The left Venn Diagram was the intersection of the genes between demethylation sites and high expression mRNA. The right Venn Diagram for the intersection of the genes between hypermethylation sites and low expression mRNA.

**Additional file 4: Table S1.** Log fold change, P value and adjusted P value of differential mRNA.

**Additional file 5: Figure S4.** The Violin Diagram for mRNA expression of 10 screened genes.

**Additional file 6: Table S2.** Annotations link in UniProt database and Search strategy in Pubmed database for screened genes.

**Additional file 7: Figure S5.** The Bar Diagram for GO term enrichment analyses of 10 screened genes.

**Additional file 8: Figure S6.** The Bubble Diagram for KEGG pathway enrichment analyses of 10 screened genes.

**Additional file 9: Table S3.** Our study provides ARDS researchers with five valuable research points.

## Data Availability

All data generated and/or analysed during this study can be found in GEO database. The series of these studies were GSE32707, GSE66890, GSE10474, GSE61672 and GSE67530.

## Abbreviations

ARDS: acute respiratory distress syndrome
GEO: gene expression omnibus
MYLK: myosin light chain kinase
SM: supplemental material
DEGs: differentially expressed genes
PPI: protein–protein interaction network
GO: gene ontology
KEGG: Kyoto Encyclopaedia of Genes and Genomes
ROC: receiver operating characteristic curves
AUC: area under the curve
PI3: peptidase inhibitor 3
CX3CR1: C-X3-C motif chemokine receptor 1
FYN: FYN proto-oncogene, Src family tyrosine kinase
DUSP6: dual specificity phosphatase 6
POLB: DNA polymerase beta
SLC3A2: solute carrier family 3 member 2
PILRA: paired immunoglobin like type 2 receptor alpha
SRPK2: SRSF protein kinase 2
RNF1: ring finger protein 1
TRIM33: tripartite motif containing 33
