# Supplementary material for: DNA methylation exploration for ARDS: a multi-omics and multi-microarray interrelated analysis
Source: J Transl Med. 2019 Oct 17;17:345. doi: 10.1186/s12967-019-2090-1 (PMC6796364; doi:10.1186/s12967-019-2090-1)
Supplement: Supplementary file 4 — Additional file 4: Table S1. Log fold change, P value and adjusted P value of differential mRNA. [file 12967_2019_2090_MOESM4_ESM.doc]

**Table S1**. Log fold change, P value and adjusted P value of differential mRNA

| Gene symbol | Log fold change | P value | adjusted P value |
| --- | --- | --- | --- |
| SRPK2 | -0.23904 | 4.17E-08 | 0.00027 |
| ALDH1A1 | -0.39682 | 9.54E-08 | 0.00031 |
| ATP2B1 | -0.46258 | 4.85E-07 | 0.001049 |
| CX3CR1 | -0.7075 | 2.49E-06 | 0.004043 |
| TREM1 | -0.59113 | 8.76E-06 | 0.011363 |
| UBE2K | 0.235234 | 1.97E-05 | 0.020678 |
| SLC7A1 | 0.262763 | 2.23E-05 | 0.020678 |
| PNP | 0.494816 | 3.49E-05 | 0.023361 |
| PILRA | -0.44773 | 3.50E-05 | 0.023361 |
| CYTIP | -0.22248 | 3.60E-05 | 0.023361 |
| PLAUR | -0.2922 | 4.47E-05 | 0.025863 |
| SLC3A2 | 0.121903 | 5.36E-05 | 0.025863 |
| DUSP6 | -0.39563 | 6.12E-05 | 0.025863 |
| PI3 | -1.01003 | 6.30E-05 | 0.025863 |
| SPAG9 | -0.25471 | 6.53E-05 | 0.025863 |
| TBC1D22B | 0.291774 | 6.64E-05 | 0.025863 |
| ATG9A | 0.299863 | 6.78E-05 | 0.025863 |
| TFRC | 0.51734 | 7.18E-05 | 0.025888 |
| FYN | -0.34693 | 8.06E-05 | 0.027513 |
| RNF19B | -0.27524 | 0.000103 | 0.031259 |
| POLB | -0.37848 | 0.000106 | 0.031259 |
| KYNU | -0.48628 | 0.000106 | 0.031259 |
| UTS2 | -0.41378 | 0.000111 | 0.031259 |
| SH3GL1 | 0.213987 | 0.00012 | 0.03249 |
| TLR6 | -0.26465 | 0.000126 | 0.032648 |
| OSBPL8 | -0.24962 | 0.000156 | 0.038843 |
| TRIM33 | -0.13571 | 0.000166 | 0.039931 |
| FAM117A | 0.377817 | 0.000177 | 0.041088 |
| RCBTB2 | -0.29625 | 0.000207 | 0.046346 |
