# Supplementary material for: DNA methylation exploration for ARDS: a multi-omics and multi-microarray interrelated analysis
Source: J Transl Med. 2019 Oct 17;17:345. doi: 10.1186/s12967-019-2090-1 (PMC6796364; doi:10.1186/s12967-019-2090-1)
Supplement: Supplementary file 6 — Additional file 6: Table S2. Annotations link in UniProt database and Search strategy in Pubmed database for screened genes. [file 12967_2019_2090_MOESM6_ESM.doc]

**Table S2.** Annotations link in UniProt database and Search strategy in Pubmed database for screened genes

| Gene symbol | Annotations link in UniProt database | Search strategy in Pubmed database |
| --- | --- | --- |
| CX3CR1 | https://www.uniprot.org/uniprot/P49238 | (((((((((CX3CR1[Title/Abstract]) OR C-X3-C motif chemokine receptor 1[Title/Abstract]) OR V28[Title/Abstract]) OR CCRL1[Title/Abstract]) OR GPR13[Title/Abstract]) OR CMKDR1[Title/Abstract]) OR GPRV28[Title/Abstract]) OR CMKBRL1[Title/Abstract])) AND (((((ARDS[Title/Abstract]) OR ALI[Title/Abstract]) OR Acute lung injury[Title/Abstract]) OR Acute Respiratory Distress Syndrome[Title/Abstract]) OR Adult Respiratory Distress Syndrome[Title/Abstract]) |
| DUSP6 | https://www.uniprot.org/uniprot/Q16828 | ((((((DUSP6[Title/Abstract]) OR dual specificity phosphatase 6[Title/Abstract]) OR HH19[Title/Abstract]) OR MKP3[Title/Abstract]) OR PYST1)) AND (((((ARDS[Title/Abstract]) OR ALI[Title/Abstract]) OR Acute lung injury[Title/Abstract]) OR Acute Respiratory Distress Syndrome[Title/Abstract]) OR Adult Respiratory Distress Syndrome[Title/Abstract]) |
| FYN | https://www.uniprot.org/uniprot/P06241 | ((((((FYN[Title/Abstract]) OR FYN proto-oncogene, Src family tyrosine kinase[Title/Abstract]) OR SLK[Title/Abstract]) OR SYN[Title/Abstract]) OR p59-FYN[Title/Abstract])) AND (((((ARDS[Title/Abstract]) OR ALI[Title/Abstract]) OR Acute lung injury[Title/Abstract]) OR Acute Respiratory Distress Syndrome[Title/Abstract]) OR Adult Respiratory Distress Syndrome[Title/Abstract]) |
| PILRA | https://www.uniprot.org/uniprot/Q9UKJ1 | ((((PILRA[Title/Abstract]) OR paired immunoglobin like type 2 receptor alpha[Title/Abstract]) OR FDF03[Title/Abstract])) AND (((((ARDS[Title/Abstract]) OR ALI[Title/Abstract]) OR Acute lung injury[Title/Abstract]) OR Acute Respiratory Distress Syndrome[Title/Abstract]) OR Adult Respiratory Distress Syndrome[Title/Abstract]) |
| POLB | https://www.uniprot.org/uniprot/P06746 | (((POLB[Title/Abstract]) OR DNA polymerase beta[Title/Abstract])) AND (((((ARDS[Title/Abstract]) OR ALI[Title/Abstract]) OR Acute lung injury[Title/Abstract]) OR Acute Respiratory Distress Syndrome[Title/Abstract]) OR Adult Respiratory Distress Syndrome[Title/Abstract]) |
| SRPK2 | https://www.uniprot.org/uniprot/P78362 | ((((SRPK2[Title/Abstract]) OR SRSF protein kinase 2[Title/Abstract]) OR SFRSK2[Title/Abstract])) AND (((((ARDS[Title/Abstract]) OR ALI[Title/Abstract]) OR Acute lung injury[Title/Abstract]) OR Acute Respiratory Distress Syndrome[Title/Abstract]) OR Adult Respiratory Distress Syndrome[Title/Abstract]) |
| PI3 | https://www.uniprot.org/uniprot/P19957 | (((PI3[Title/Abstract]) OR peptidase inhibitor 3[Title/Abstract])) AND (((((ARDS[Title/Abstract]) OR ALI[Title/Abstract]) OR Acute lung injury[Title/Abstract]) OR Acute Respiratory Distress Syndrome[Title/Abstract]) OR Adult Respiratory Distress Syndrome[Title/Abstract]) |
| RNF19B | https://www.uniprot.org/uniprot/Q6ZMZ0 | (((RNF19B[Title/Abstract]) OR ring finger protein 19B[Title/Abstract])) AND (((((ARDS[Title/Abstract]) OR ALI[Title/Abstract]) OR Acute lung injury[Title/Abstract]) OR Acute Respiratory Distress Syndrome[Title/Abstract]) OR Adult Respiratory Distress Syndrome[Title/Abstract]) |
| TRIM33 | https://www.uniprot.org/uniprot/Q9UPN9 | (((TRIM33[Title/Abstract]) OR tripartite motif containing 33[Title/Abstract])) AND (((((ARDS[Title/Abstract]) OR ALI[Title/Abstract]) OR Acute lung injury[Title/Abstract]) OR Acute Respiratory Distress Syndrome[Title/Abstract]) OR Adult Respiratory Distress Syndrome[Title/Abstract]) |
| SLC3A2 | https://www.uniprot.org/uniprot/P08195 | (((SLC3A2[Title/Abstract]) OR solute carrier family 3 member 2[Title/Abstract])) AND (((((ARDS[Title/Abstract]) OR ALI[Title/Abstract]) OR Acute lung injury[Title/Abstract]) OR Acute Respiratory Distress Syndrome[Title/Abstract]) OR Adult Respiratory Distress Syndrome[Title/Abstract]) |
