# Supplementary material for: DNA methylation exploration for ARDS: a multi-omics and multi-microarray interrelated analysis
Source: J Transl Med. 2019 Oct 17;17:345. doi: 10.1186/s12967-019-2090-1 (PMC6796364; doi:10.1186/s12967-019-2090-1)
Supplement: Supplementary file 9 — Additional file 9: Table S3. Our study provides ARDS researchers with five valuable research points. [file 12967_2019_2090_MOESM9_ESM.doc]

**Table S3. Our study provides ARDS researchers with five valuable research points**

| 1 | 22654 hypermethylation sites and 21785 demethylation sites may exist in ARDS patients |
| --- | --- |
| 2 | 29 differential mRNAs may exist between ARDS patients and healthy people |
| 3 | In ARDS patients, 32 hypermethylation sites regulated 12 genes resulting in their mRNA low expression and that 8 demethylation sites regulated 4 genes resulting in their mRNA high expression |
| 4 | 30 DNA methylation sites may regulate transcription of 10 genes to cause ARDS |
| 5 | ROC analyses showed the area under the curve (AUC) of cg03341377, cg24310395, cg07830557 and cg08418670 was up to 0.99, which meant that these DNA methylation alterations were promising biomarkers for the diagnosis of ARDS. |
